# Associations of Tumor PD-1 Ligands, Immunohistochemical Studies, and Textural Features in ^18^F-FDG PET in Squamous Cell Carcinoma of the Head and Neck

**DOI:** 10.1038/s41598-017-18489-2

**Published:** 2018-01-08

**Authors:** Rui-Yun Chen, Ying-Chun Lin, Wei-Chih Shen, Te-Chun Hsieh, Kuo-Yang Yen, Shang-Wen Chen, Chia-Hung Kao

**Affiliations:** 1 Department of Pathology, China Medical University Hospital, Taichung, Taiwan; 2 Department of Radiation Oncology, China Medical University Hospital, Taichung, Taiwan; 3 The Ph.D. Program for Cancer Biology and Drug Discovery, China Medical University and Academia Sinica, Taichung, Taiwan; 4 Department of Computer Science and Information Engineering, Asia University, Taichung, Taiwan; 5 Department of Nuclear Medicine and PET Center, China Medical University Hospital, Taichung, Taiwan; 6 Department of Biomedical Imaging and Radiological Science, China Medical University, Taichung, Taiwan; 7 School of Medicine, China Medical University, Taichung, Taiwan; 8 School of Medicine, Taipei Medical University, Taipei, Taiwan; 9 Graduate Institute of Clinical Medical Science, School of Medicine, College of Medicine, China Medical University, Taichung, Taiwan; 10 Department of Bioinformatics and Medical Engineering, Asia University, Taichung, Taiwan

## Abstract

To know tumor PD-L1 expression through IHC or the FDG-PET related radiomics, we investigated the association between programmed cell death protein 1 ligand (PD-L1) expression and immunohistochemical (IHC) biomarkers or textural features of 18F-fluoro-2-deoxdeoxyglucose positron emission tomography (^18^F-FDG PET) in 53 oropharyngeal or hypopharyngeal cancer patients who were ready to undergo radiotherapy-based treatment. Differences in textural features or biomarkers between tumors with and without PD-L1 expression were tested using a Mann–Whitney U test. The predicted values for PD-L1 expression were examined using logistic regression analysis. The mean percentages of tumor PD-L1 expression were 6.2 ± 13.5. Eighteen tumors had PD-L1 expression ≥5%, whereas 30 tumors ≥1%. Using a 5% cutoff, the *p16* staining percentage and the textural index of correlation were two factors associated with PD-L1 expression. The odds ratios (ORs) were 17.00 (*p* = 0.028) and 0.009 (*p* = 0.015), respectively. When dichotomizing PD-L1 at 1%, the *p16* and *Ki-67* staining percentages were two predictors for PD-L1 expression with ORs of 11.41 (*p* = 0.035) and 757.77 (*p* = 0.045). *p16* and *Ki-67* staining percentages and several PET/CT-derived textural features can provide supplemental information to determine tumor PD-L1 expression in HNCs.

## Introduction

Head and neck cancer (HNC) is the fifth most common malignancy worldwide^1^, with most patients presenting with locoregionally advanced disease, and more than 50% experiencing recurrence within 3 years^2^. Advances in the understanding of the immune system’s role in tumor development have led to malignant cells being recognized as having the ability to elude immune control through the deregulation of inhibitory signals or other specific immune checkpoints. A randomized trial investigating the inhibitors of programmed cell death protein 1 (PD-1) in recurrent HNC squamous cell carcinoma showed promising results^3^. Tumor PD-1 ligand (PD-L1) expression reflects an immune-active microenvironment; although it is associated with other immunosuppressive molecules including PD-1 and PD-L2, PD-L1 expression is the single factor most closely correlated with response to anti-PD-1 blockade^4^. Although no standardized method for PD-L1 detection or cutoff has been defined, significant associations were found between tumor PD-L1 expression, the presence of intratumoral immune cell infiltrates, and the expression of PD-1 receptor in tumor-infiltrating lymphocytes (TILs). This suggested that PD-L1 reflects an immune-reactive milieu^4^. In addition, recent data suggested that patients with tumors that overexpress PD-L1 have an increased chance of superior clinical outcomes after anti-PD-1–directed therapy^5^. To date, other than quantifying PD-L1 through immunohistochemistry (IHC), no predictors are available to identify patients with tumors that have a higher PD-L1 expression and may benefit from checkpoint inhibitors.

Among image-based modalities for noninvasive tumor assessment, positron emission tomography with 18-fluorodeoxyglucose (^18^F-FDG PET) remains one of the most extensively used methods in the diagnostic workup of patients with various cancers. Recently, a pilot study for nonsmall cell lung cancer showed a direct association between metabolic parameters on FDG-PET and the expression of tumor-related immunity markers, suggesting a potential role for FDG-PET in characterizing the tumor microenvironment and selecting suitable candidates as checkpoint inhibitors^6^. However, no studies have reported the correlation between metabolic information on FDG-PET and the tissue expression of PD-L1 across various cancers. Because of the intrinsically dynamic nature of immune responses, and given the hurdles of performing new tumor biopsies aimed at investigating potential molecular predictors of checkpoint inhibitor activity in pretreated HNCs, noninvasive or minimally invasive approaches to aid patient selection are warranted. Moreover, the microenvironments of cancer tissues often present with biological heterogeneity corresponding to hypoxia, angiogenesis, or immunogenicity^7^. Such biological characteristics are of interest because they are associated with radiomics^8^. To gain greater knowledge regarding the effect of a tumor microenvironment, detected through IHC or the FDG-PET related radiomics, on tumor PD-L1 expression, we conducted this study to investigate the associations between tumor PD-L1 expression, several protein biomarkers involving the pathogenesis of hypoxia, angiogenesis, and proliferation, and ^18^F-FDG PET-based textural features in patients with HNCs.

## Materials and Methods

### Study population

Between January 2007 and December 2013, we retrospectively analyzed 53 patients with newly diagnosed oropharyngeal or hypopharyngeal squamous cell carcinoma who were ready to receive definitive chemoradiotherapy or radiotherapy for organ preservation at China Medical University Hospital. All patients had undergone pretreatment ^18^F-FDG PET-computed tomography (PET/CT) for staging. All patients had normal serum glucose levels before undergoing PET/CT. This study was approved by a local institutional review board (CMUH103-REC2-093FR and DMR99-IRB-010-1). The IRB also specifically waived the consent requirement.

### Immunohistochemistry

As described previously using IHC^9^, gene expression profiles can be classified into hypoxic markers (*Glut1*, *CAIX*, *VEGF*, and *HIF-1α*)^10^, radioresistant markers (*Bcl-2*, *CLAUDIN-4*, *YAP-1*, and *c-Met*)^11^, a proliferative marker (*Ki-67*)^12^, a tumor progression factor (*EGFR*)^13^, and a surrogate marker for human papillomavirus (HPV; *CDKN2A*)^14^. The spots of each pretreatment incisional biopsy were microscopically selected and arranged pairwise in tissue microarray blocks. Each tumor was represented by one tissue core on a tissue microarray. Furthermore, 4-µm-thick paraffin sections were deparaffinized and microwaved according to standard procedures before being processed for IHC staining.

Tissue slides were scored by two pathologists blinded to the study endpoints, and any disagreement between the two observers was resolved through consensus. This was because data dichotomization in IHC studies could distort the exact correlation between the study endpoints and protein biomarkers. Continuous scoring of the biomarkers using an H-score were reported, which was derived through a summation of the percentage of area stained at each intensity level multiplied by the weighted intensity. As detailed previously^9^, nuclear staining was performed for *HIF-1α* and *Ki*-67, whereas only cell membrane staining was performed for *EGFR*, *CAIX*, *c-Met*, *Claudin-4*, and *Glut-1*. *VEGF* and *Bcl-2* exhibited a membranous or cytoplasmic staining pattern. *YAP-1* was visualized through cytoplasmic or nuclear staining.

### PD-L1 expression detected through IHC

Tumor PD-L1 biomarker was evaluated through IHC staining using DAKO clone 22C3 pharmDx (DAKO, Carpinteria, CA). Formalin-fixed paraffin embedded tumor tissues were deparaffinized and dehydrated in xylene and graded ethanol solutions. PD-L1 expressions were scored according to a tumor proportion score, which was defined as the percentage of tumor cells with complete or partial membranous staining at any intensity^15,16^. Representative images of IHC staining for PD-L1 are illustrated in Appendix 1.

### HPV status determination using p16 as a marker

In this study, *p16* overexpression detected through IHC staining was considered a surrogate marker for HPV involvement, which was verified in ten patients through the detection of HPV DNA using chromogenic *in situ* hybridization and polymerase chain reaction. Moreover, *p16* expression was scored as positive when strong and diffuse nuclear and cytoplasmic staining was present in ≥70% of tumor cells^14^. Representative images of *p16* positive cells are illustrated in Appendix 2. Fourteen patients (26%) were identified as having HPV-associated cancers. Tumors originated in the oropharynx and hypopharynx in 30 and 23 patients, respectively. The median age of the patients was 51 years. We performed tumor staging according to the American Joint Committee on Cancer criteria and observed that 7 and 46 patients had stage III and IVA-IVB cancer, respectively. Patient characteristics are listed in Table 1.

**Table 1:** Patients’ characteristics (N = 53).

Variables	Value
Age (year)	median 53 (range, 32 ~ 75)
Gender	Male: 53
Primary tumor site	
orapharynx	30 (57%)
hypopharynx	23 (43%)
T stage	
T1	1(2%)
T2	22 (41%)
T3	18 (34%)
T4	12 (23%)
AJCC stage	
III	7 (13%)
IVA	46 (87%)
Histology grade of squamous cell carcinoma	
well differentiated	14 (26%)
moderately differentiated	17(32%)
poorly differentiated	11 (21%)
unclassified	11 (21%)
Smoking	
smoker	45(85%)
never-smoker	8(15%)
Betel nut squid	
yes	41(77%)
never	12 (23%)
Alcohol drinking	
alcoholism	36 (68%)
non-alcoholism	17 (32%)
HPV status	
p16 expression ≥70%	14 (26%)
p16 expression <70%	39 (74%)
SUVmax	mean 10.1 ± 4.8 (range, 2.3~ 24.1)
CT-based tumor volume (ml)	mean 32.8 ± 33.7 (range, 3 ~ 375)

AJCC = American Joint Committee on Cancer criteria.

### PET/CT imaging

All patients were scanned using a PET/CT scanner (PET/CT-16 slice, Discovery STE; GE Medical System, Milwaukee, WI, USA). The patients were requested to fast for at least 4 hours before the administration of (^18^F)-FDG, and FDG PET/CT imaging was conducted approximately 60 minutes after the administration of 370 MBq of ^18^F-FDG. Thus, FDG uptake was determined in order to calculate the standardized uptake value (SUV). The maximum SUV (SUV_max_) was confirmed through consensus between two nuclear medicine physicians.

In addition, the CT-based gross tumor volume was obtained for all patients as previously described^9^.

### Calculation of textural indices

The metabolic tumor volume (MTV) of a tumor was delineated through an adaptive threshold method using a signal to background ratio. A voxel was defined as a local maximum if its SUV was not smaller than those of its neighbors. The SUV_max_ of a tumor was the local maximum with the largest SUV within the spatial extent. All voxels with an SUV greater than SUV_max_ × 0.7 that were connected to the SUV_max_ were grouped to form a temporary MTV of the tumor. Similarly, the local maximum located outside of the temporary MTV with the shortest distance was identified and used to delineate a background MTV in the same manner. The average SUV of the temporary MTV, SUV_mean_, and that of the background MTV, SUV_BKG_, were calculated and used to define an adaptive threshold as follows:

$${\rm{Threshold}}={\rm{\beta }}\,{{\rm{SUV}}}_{{\rm{mean}}}+{{\rm{SUV}}}_{{\rm{BKG}}}$$

where β = 0.15^17^. Finally, the MTV of the tumor was delineated using the threshold.

The heterogeneity of a tumor was evaluated using its textural features. The SUVs within a tumor were discretized using fixed bin widths of 0.05, 0.1, 0.2, 0.25, and 0.5 g/mL. For each discretization, four matrices were calculated to describe the texture of SUVs within the tumor: the gray-level co-occurrence matrix (GLCM)^18^, neighboring gray-level dependence matrix^19^, gray-level run length matrix (GLRLM)^20^, and gray-level size zone matrix (GLSZM)0^21^. Because the definitions of GLCM and GLRLM are directional, 13 matrices were calculated for all possible orientation settings for each matrix. Finally, the textural features defined for each matrix were calculated. In total, 41 textural features or histograms were extracted (Appendix 3). The features were adopted on the basis of their ability to predict local recurrence after definitive radiotherapy or chemoradiotherapy, as previously described^8^.

### Statistical analysis

Correlations between PD-L1 expression and different IHC studies or textural features were examined using Spearman’s rank correlation coefficient, with the alpha level set at 0.01. To compare their predictive ability for PD-L1 expression, all of the extracted features were first examined through receiver-operating characteristic (ROC) curve analysis using the aforementioned discretization methods. The abilities to predict mutational status were compared through examining the area under the curve (AUC). The optimal discretization method for each feature was chosen and entered for further analysis. If the AUC for PD-L1 expression was statistically significant, the quantitative differences for these indices or IHC studies between tumors with and without PD-L1 expression were examined using a Mann–Whitney U test. Thereafter, all of the statistically significant textural indices combined with IHC parameters were tested with logistic regression analysis to seek the independent predictable factors for PD-L1 expression. All analyses were two-sided, and *p* < 0.05 was considered statistically significant. For statistically significant textural features associated with PD-L1 expression, we determined the optimal cutoff by the couple sensibility–specificity using ROC analysis. Statistical analyses were performed using SPSS, version 16.0 (SPSS Inc, Chicago, IL, USA).

### Ethical approval

All procedures performed in studies involving human participants were in accordance with the ethical standards of the institutional and/or national research committee and with the 1964 Helsinki declaration and its later amendments or comparable ethical standards.

### Informed consent

The IRB also specifically waived the consent requirement.

## Results

### Correlations between PD-L1 expression and IHC studies or textural features

The four groups of textural indices, the conventional PET-related parameters, the histograms, and the IHC staining intensities of the biomarkers were all retrieved for the entire cohort. The mean percentage of tumor PD-L1 expression was scored (6.2 ± 13.5, range: 0–75). With different cutoffs, 12, 18, and 30 tumors had PD-L1 expressions of ≥10%, ≥5%, and ≥1%, respectively.

The PD-L1 expressions were positively correlated with *Ki-67 (p* = 0.003, γ = 0.40), *c-Met (p* = 0.015, γ = 0.33), and *p16* (*p* = 0.001, γ = 0.43). The three IHC biomarkers were not related to each other (*Ki-67* and *c-Met* [*p* = 0.62, γ = 0.07], *Ki-67* and *p16* [*p* = 0.08, γ = 0.25], and *c-Met* and *p16* [*p* = 0.06, γ = 0.26]). With *p16* expression ≥70% as a surrogate of HPV infection, the proportion of tumors having PD-L1 expressions of ≥5% and ≥1% was 50% and 71% in patients with HPV-positive tumors (N = 14) and 28% and 51% in those with HPV-negative tumors (N = 39).

For the various textural features, PD-L1 expression intensity was inversely correlated with gray-level nonuniformity for run (GLNUr; *p* = 0.04, γ = −0.27), run percentage (RP; *p* = 0.03, γ = −0.30), and short-zone low gray-level emphasis (SZLGE; *p* = 0.04, γ = −0.28).

In addition, the Mann–Whitney U test revealed that tumors from nonsmokers had a higher expression of PD-L1 and *p16* levels, at *p* = 0.003 and *p* = 0.004, respectively.

### Comparison of the predictive ability of different IHC studies and textural indices for PD-L1 expression

Because few tumors had PD-L1 expressions of ≥10%, a binary classification of PD-L1 expression using this cutoff was excluded from the analysis. Table 2 summarizes the predictive abilities across various protein biomarkers or textural indices for PD-L1 expression using cutoffs of 1% and 5%. The ROC curves indicated that *Ki-67*, *p16*, and several textural indices were predictive of PD-L1 expressions of ≥5%. These textural features included correlation, entropy, and energy from GLCM (26-connected and bin width = 0.1); short-run emphasis (SRE), long-run emphasis (LRE), run length nonuniformity (RLNU), GLNUr, and RP from GLRLM (26-connected and bin width = 0.5); coarseness and contrast from NGLDM (18-connected and bin width = 0.2); and gray-level nonuniformity for zone (GLNUz) and zone length nonuniformity (ZLNU) from GLSZM (18-connected and bin width = 0.5). When dichotomizing the PD-L1 expression levels at 1%, the ROC curves revealed that the good predictive performance of *Ki-67* and *p16*. However, SZLGE became a sole feature for PD-L1 expression among the indices.

**Table 2:** Predictive abilities of biomarkers or textural indices for PD-L1 expression according to 1% and 5% cutoffs.

Classification of matrix	Index	AUC/*p* valuecutoff 5%	AUC*/p* value.cutoff 1%
Immunohistochemical biomarker	*Ki-67*	0.69 ± 0.07/0.024	0.72 ± 0.07/0.006
	*p16*	0.72 ± 0.07/0.011	0.75 ± 0.07/0.002
Conventional PET-related parameter	SUV_max_	0.50 ± 0.09/0.98	0.66 ± 0.08/0.05
	MTV	0.28 ± 0.07/0.009	0.45 ± 0.08/0.53
	TLG_mean_	0.32 ± 0.08/0.035	0.52 ± 0.08/0.79
Gray Level Cooccurrence Matrix (GLCM)	correlation	0.24 ± 0.07/0.002	0.45 ± 0.08/0.53
	energy	0.69 ± 0.08/0.035	0.56 ± 0.08/0.48
	entropy	0.31 ± 0.08/0.029	0.46 ± 0.08/0.58
Gray-Level Run Length Matrix (GLRLM)	SRE	0.71 ± 0.07/0.011	0.56 ± 0.08/0.45
	LRE	0.29 ± 0.07/0.011	0.43 ± 0.08/0.40
	GLNUr	0.28 ± 0.08/0.011	0.41 ± 0.08/0.25
	RP	0.29 ± 0.07/0.011	0.39 ± 0.08/0.18
	RLNU	0.30 ± 0.08/0.017	0.46 ± 0.08/0.66
Neighborhood Gray-Level Different Matrix (NGLDM)	coarseness	0.68 ± 0.08/0.038	0.51 ± 0.08/0.89
	contrast	0.70 ± 0.08/0.021	0.61 ± 0.08/0.20
Gray-Level Zone Length Matrix (GLSZM)	GLNUz	0.28 ± 0.07/0.011	0.45 ± 0.08/0.54
	ZLNU	0.33 ± 0.08/0.044	0.52 ± 0.08/0.84
	SZLGE	0.35 ± 0.08/0.08	0.31 ± 0.07/0.018

SRE = short-run emphasis; LRE = long-run emphasis; LGRE = low gray-level run emphasis; HGRE = high gray-level run emphasis; SRLGE = short-run low gray-level emphasis; SRHGE = short-run high gray-level emphasis; LRLGE = long-run low gray-level emphasis; LRHGE = long-run high gray-level emphasis; GLNUr = gray-level nonuniformity for run; RLNU = run length nonuniformity; RP = run percentage; SZE = short-zone emphasis; LZE = long-zone emphasis; LGZE = low gray-level zone emphasis; HGZE = high gray-level zone emphasis; SZLGE = short-zone low gray-level emphasis; SZHGE = short-zone high gray-level emphasis; LZLGE = long-zone low gray-level emphasis; LZHGE = long-zone high gray-level emphasis; GLNUz = gray-level nonuniformity for zone; ZLNU = zone length nonuniformity; and ZP = zone percentage.

**Figure 1:**
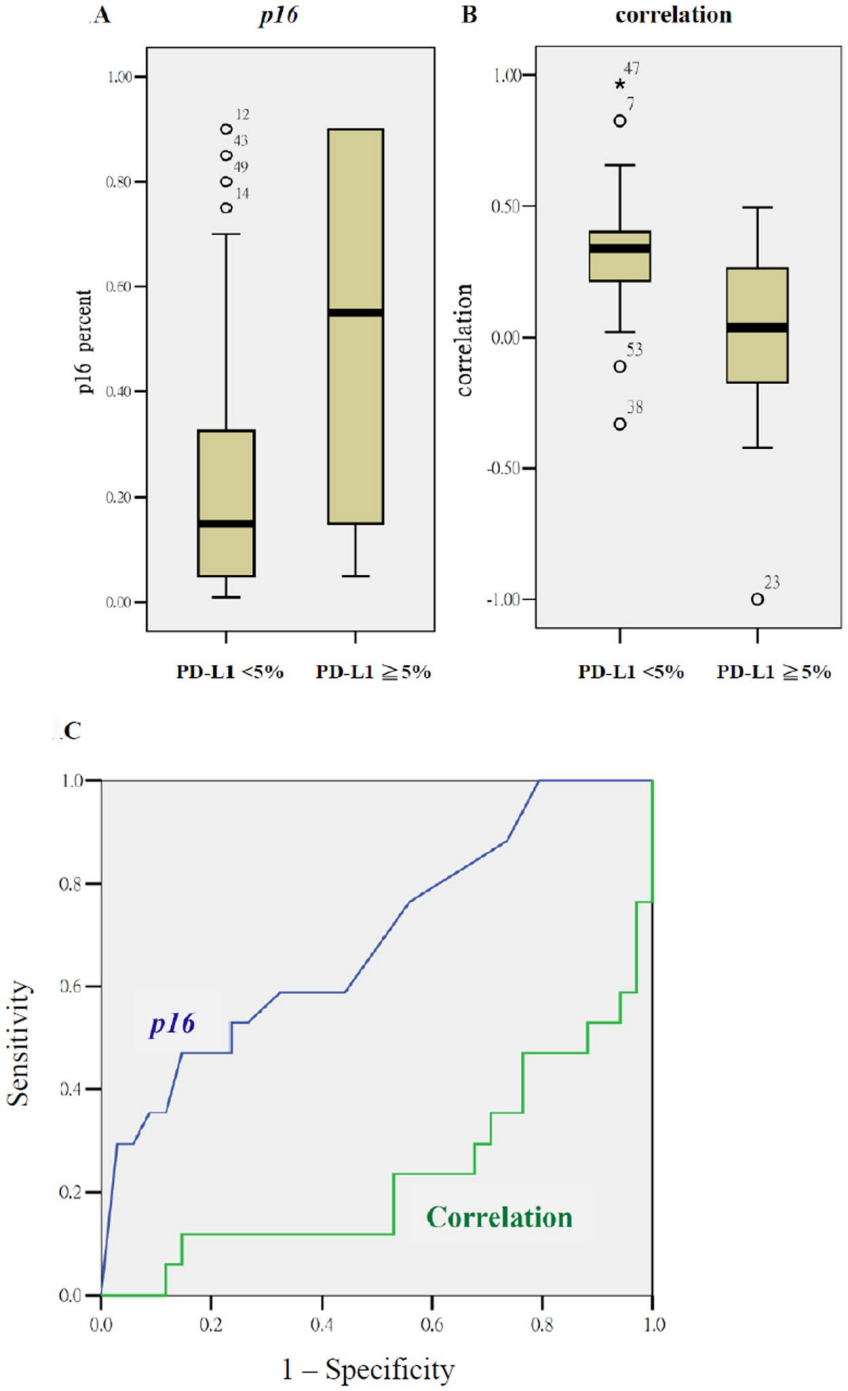
PD-L1 expression of ≥5% associated with quantitative values of the IHC intensity of *p16* (1 A), textural index of correlation (IB), and their ROC curves (1 C). The AUCs for *p16* and the textural index of correlation were 0.72 ± 0.07 (*p* = 0.011) and 0.24 ± 0.07 (*p* = 0.002), respectively.

The quantitative differences are listed in Table 3. The Mann–Whitney U test showed that *Ki-67*, *p16*, and 12 textural indices were predictive for PD-L1 expressions of ≥5%. The logistic regression analysis indicated that the *p16* staining percentage and correlation from GLCM were two independent predictors for PD-L1 expression. The odds ratios (ORs) were 17.00 (*p* = 0.028; 95% confidence interval [CI]: 1.35–214.52) and 0.009 (*p* = 0.015; 95% CI: 0.00–0.41), respectively. Figure 1 depicts the quantitative difference of *p16* and correlation from GLCM between tumors with PD-L1 expressions of ≥5% and < 5%, as well as the corresponding ROC curves. The mean percentage intensity of *p16* for tumors with PD-L1 expressions of ≥5% and < 5% were 50.28 ± 36.56% and 25.91 ± 27.38%, whereas the values of correlation from GLCM were −0.04 ± 0.45 and 0.31 ± 0.24 for the two groups, respectively.

**Table 3:** Mann-Whitney U test for various patient-, tumor- and texture features according to tumor expression of PD-L1 ≧5%.

Variables	PD-L1 ≧ 5% (N = 18)	PD-L1 < 5% (N = 35)	*p* value
T3-T4 tumor	9/18	21/35	0.49
Smoking	13/18	32/35	0.06
CT-based tumor volume (ml)	21.37 ± 21.94	38.67 ± 63.79	0.20
*Ki-67*(%)	21.33 ± 14.95	13.51 ± 10.81	0.005
*p16* (%)	50.28 ± 36.56	25.91 ± 27.38	0.002
SUVmax	10.07 ± 4.62	10.18 ± 4.89	0.99
MTV (ml)	7.41 ± 10.79	92.49 ± 429.07	0.009
TLGmean (g)	59.36 ± 98.52	269.62 ± 892.22	0.035
correlation (GLCM)	−0.04 ± 0.45	0.31 ± 0.24	0.002
energy (GLCM)	0.09 ± 0.15	0.03 ± 0.06	0.018
entropy (GLCM)	3.50 ± 1.53	4.64 ± 1.36	0.015
coarseness (NGLDM)	0.11 ± 0.12	0.05 ± 0.05	0.014
contrast (NGLDM)	0.26 ± 0.65	0.20 ± 0.95	0.021
SRE	0.98 ± 0.04	0.94 ± 0.06	0.01
LRE	1.09 ± 0.14	1.28 ± 0.36	0.01
RP	1.02 ± 0.96	2.84 ± 4.52	0.011
RLNU	65.35 ± 90.67	464.92 ± 1830.62	0.017
GLNUr	9.99 ± 11.83	104.64 ± 463.76	0.011
GLNUz	3.63 ± 3.34	10.78 ± 26.75	0.011
ZLNU	12.44 ± 14.63	36.39 ± 79.97	0.044

SRE = short-run emphasis; LRE = long-run emphasis; LGRE = low gray-level run emphasis; HGRE = high gray-level run emphasis; SRLGE = short-run low gray-level emphasis; SRHGE = short-run high gray-level emphasis; LRLGE = long-run low gray-level emphasis; LRHGE = long-run high gray-level emphasis; GLNUr = gray-level nonuniformity for run; RLNU = run length nonuniformity; RP = run percentage; SZE = short-zone emphasis; LZE = long-zone emphasis; LGZE = low gray-level zone emphasis; HGZE = high gray-level zone emphasis; SZLGE = short-zone low gray-level emphasis; SZHGE = short-zone high gray-level emphasis; LZLGE = long-zone low gray-level emphasis; LZHGE = long-zone high gray-level emphasis; GLNUz = gray-level nonuniformity for zone; ZLNU = zone length nonuniformity; and ZP = zone percentage.

Note: 1. Immunohistochemical intensity and textural features are expressed as means ± standard deviation.

2. T-stage and smoking were examined by Chi-square test.

**Figure 2:**
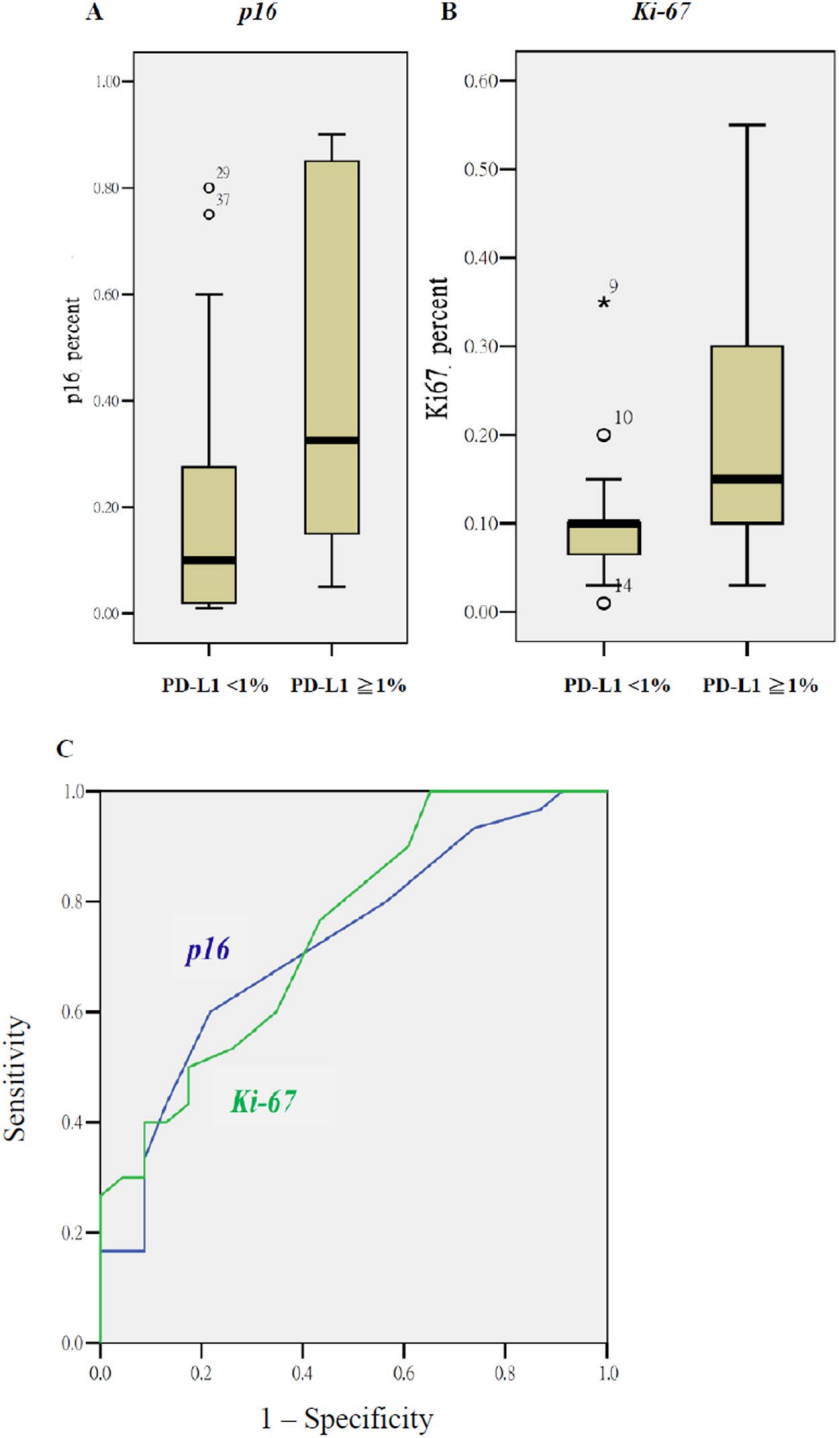
PD-L1 expression of ≥1% associated with the quantitative values of the IHC intensity of *p16* (1 A), Ki-67 (IB), and their ROC curves (1 C). The AUCs for *p16* and the textural index of correlation were 0.75 ± 0.06 (*p* = 0.002) and 0.72 ± 0.07 (*p* = 0.006), respectively.

When dichotomizing PD-L1 expression at 1%, the logistic regression analysis revealed that the *p16* and *Ki-67* staining percentages were two predictors for PD-L1 expression. The ORs were 11.41 (*p* = 0.035; 95% CI: 1.19–109.10) and 757.77 (*p* = 0.045; 95% CI: 1.18–487860.18), respectively. The quantitative difference of the two biomarkers between tumors with PD-L1 expression of ≥1% and < 1% are illustrated in Fig. 2. None of the PET/CT parameters or textural features were independent factors for PD-L1 expression. Smoking, tumor origin, and advanced T- or N-staging did not affect the PD-L1 expression with cutoffs of 1% or 5%.

### Accuracy in predicting PD-L1 expression

Based on the aforementioned predictive parameters, we attempted to determine the optimal cutoffs for the biomarkers to distinguish tumors above and below the cutoffs of PD-L1 expression. When a cutoff value for *p16* of 15% was chosen, the sensitivity, specificity, and accuracy for predicting PD-L1 expression of ≥5% were 78%, 46%, and 57%, respectively. If the cutoff for PD-L1 expression was 1%, the corresponding values were 77%, 57%, and 68%, respectively.

Moreover, correlation from GLCM was a negative predictor for PD-L1 expression of ≥5% (AUC = 0.24). When using an optimal cutoff of 0.26%, the sensitivity, specificity, and accuracy of predicting PD-L1 expression of < 5% were 66%, 67%, and 66%, respectively.

### Correlation and predictive ability of IHC studies and textural features

Except for *YAP1* and *EGFR*, correlation was observed between the studied biomarkers and certain textural features (Appendix 4). Several textural features from GLCM and NGLDM were correlated well with *Claudin-4* levels, and others were related to hypoxic markers such as *VEGF* or *HIF-1α*. Appendix 5 shows the quantitative values of textural indices that were associated with the intensity of biomarkers according to the 50th percentile of the IHC score, or the aforementioned optimal cutoffs^9^. In summary, the overexpression of hypoxic markers *(VEGF*, *HIF-1α)* was positively associated with the values of entropy (GLCM), GLNUz, and RLNU. In addition, high gray-level run emphasis (HGRE), short-run high gray-level emphasis (SRHGE), high gray-level zone emphasis (HGZE), and short-zone high gray-level emphasis (SZHGE) were all positively related to *VEGF*, *Glut1*, and *Ki-67* levels, but negatively related to *Claudin-4* and *c-Met*. Furthermore, contrast (GLCM) and dissimilarity were positively associated with *VEGF and Glut1* levels, but negatively related to *Claudin-4* and *c-Met*. When using a cutoff of 30% for *CAIX* expression, homogeneity and variance were related to *CAIX* level.

## Discussion

Immunotherapy represents a paradigm shift in cancer treatment; checkpoint inhibitors targeting the PD-1/PD-L1 axis have been reported to provide promising clinical responses in patients with various types of cancer^5,22^. Although PD-L1 expression in numerous types of tumor has increased the chance of clinical outcomes in some cancers with anti-PD-1–directed therapy, the lack of a clear definition of positive tumor PD-L1 staining through IHC is problematic. Cutoffs for a positive result range from > 1% to > 50% based on the percentage of tumor cells stained, which suggests a connection with the existing issue of PD-L1 expression heterogeneity within the microenvironment^5^. By integrating several protein biomarkers and FDG-PET-based textural features, this pilot study revealed the association between tumor PD-L1 expression and tumor microenvironments or radiomics. Although IHC study for PD-L1 expression remains a standard care option, imaging studies or adjunctive biomarkers can provide additional information to circumvent the dynamic nature of immune microenvironments, or when timely biopsy cannot be employed.

The ratio of tumor cell PD-L1 expression in our cohort was compatible with other HNC clinical trials^3,23^, with a positive detection rate of 20–35% obtained through IHC with a 5% cutoff. In addition, the PD-L1 expression according to HPV status was also in agreement with that of a molecular study^24^, which reported that 70% of HPV-associated and 29% of non-HPV-associated HNCs had PD-L1 expression at the same cutoff. Theoretically, PD-L1 is biologically active only when expressed on the cell membrane, either through dynamic IFN-γ expression or constitutive oncogene activation^5,25^. Oncogene-driven PD-L1 expression is characterized by the lack of an immune infiltrate^4^. Several studies have demonstrated the associated pathways related to oncogene activation, including *PTEN* loss^26,27^, *EGFR* activation^28^, and tumor hypoxia^29^. By contrast, a positive link between tumor *p16* and PD-L1 expression in our study highlighted the model through which IFN-γ and other cytokines associated with an immune response can induce PD-L1 in tumor cells^24^. Therefore, in patients with HPV-associated HNC, our data (as well as two other studies) suggest a rationale for the therapeutic blockade of the PD-1/PD-L1 pathway^3,24^. Moreover, two additional studies have investigated the prognostic role of PD-L1 and other clinicopathologic features in patients with breast cancer^30,31^ and revealed that tumor PD-L1 expression is associated with an increased proliferation index of *Ki-67*. These findings were also observed in our data using the HNC cohort, although few biological studies have explored the molecular mechanism linking *Ki-67* and the PD-L1 expression pathway. Therefore, more studies across various cancers are required to clarify the biological process.

Lopci *et al*.^6^ conducted a pilot study to examine the associations between FDG-PET and the immune-active features of the tumor microenvironment. They found significant correlations between SUV_max_ and SUV_mean_ and the expressions of CD8-TILs and PD-1-TILs. However, no close relationship existed between the metabolic parameters and tumor cell PD-L1 expression. By using comprehensive FDG-PET-related textural features, the current study was the first to indicate that metabolic imaging phenotypes are able to predict tumor PD-L1 expression. To maximize predictive accuracy, a large sample size combined with machine learning processes for the selection of the discretization method may facilitate optimization of the performance of the textural features. If our findings are reproducible in additional validation studies, metabolic imaging features can be implemented to provide additional information if biopsies cannot be performed.

In addition, this study was the first to suggest that metabolic textural features from FDG-PET can indicate characteristics of some tumor microenvironments including hypoxia, angiogenesis, radioresistance, and tumor proliferation. To date, because no robust evidence exists to definitively dichotomize these protein biomarkers by differentiating treatment outcomes, we categorized them according to the 50th percentile or the optimal cutoffs reported in our previous study. In particular, several textural features were associated with tumor hypoxic markers. In oncology, identifying intratumoral areas with hypoxia is crucial because several studies on HNCs have concluded that partial oxygen pressure is one of the strongest independent predictors of overall survival, regardless of the treatment modality^32^. Although one study reported that there is an association between SUV_max_ of FDG and ^18^F-labeled nitroimidazoles (FMISO) in head and neck tumors^33^, future prospective studies are required to compare FMISO and textural features of FDG to image the tumor hypoxia. Currently, little evidence exists to support a straightforward correlation between textural heterogeneity and any specific underlying physiological processes or biological heterogeneity, our findings suggest that future studies could clarify the molecular mechanisms that may be related to the interplay between imaging phenotypes and tumor microenvironments.

The findings of this study should be interpreted cautiously because of the small sample size and retrospective study design. External validation studies using an independent dataset with similar imaging and IHC studies are necessary to confirm these findings. The results would have been more robust if more immune features from tumor microenvironments, such as CD8-TILs, PD-1-TILs, and CD68 tumor-associated macrophages, were included under the scope of our analysis. However, when tumor PD-L1 expression was observed, it was frequently associated with infiltrating immune cells^4^. To elucidate the cross-talk between various immune parameters or cells, the use of larger tissue specimens instead of paraffin embedded tumor tissues is essential to recruit more immune-active markers. Furthermore, our study could not imply the correlation of FDG-PET radiomics or IHC biomarkers and the therapeutic effects with anti-PD-1 or anti-PD-L1 antibodies since the PD-L1 expression does not entirely associate with the therapeutic effects. Therefore, textural features in ^18^F-FDG-PET should be investigated as potential biomarkers for checkpoint inhibitors. Finally, features derived from FDG-PET-CT or IHC biomarkers remain insufficient to replace IHC testing for PD-L1 because predictive specificity and accuracy were not completely acceptable. To maximize their supplemental roles, a combination with various features or more biomarkers should be tested as a potential approach. Nonetheless, our results provide an initial step to link imaging phenotypes or IHC biomarkers and immune-active landscapes in order to maximize therapeutic strategies for checkpoint inhibitors.

## Conclusion

In patients with HNC, *p16* and *Ki-67* staining percentages detected using IHC and several ^18^F-FDG PET/CT-derived textural features can provide supplemental information to determine tumor PD-L1 expression. The PD-L1 expressions were positively correlated with *p16* and *Ki-67*, whereas the textural index of correlation was a negative predictor for PD-L1 expression of ≥5%. Further studies are required to validate our findings and to maximize the predictive accuracy.

## Electronic supplementary material

Appendix
