# Supplementary material for: Associations of Tumor PD-1 Ligands, Immunohistochemical Studies, and Textural Features in 18F-FDG PET in Squamous Cell Carcinoma of the Head and Neck
Source: Sci Rep. 2018 Jan 8;8:105. doi: 10.1038/s41598-017-18489-2 (PMC5758832; doi:10.1038/s41598-017-18489-2)
Supplement: Supplementary file 1 — Appendix [file 41598_2017_18489_MOESM1_ESM.pdf]

Associations of Tumor PD-1 Ligands, Immunohistochemical Studies, and Textural Features in ^{18}F-FDG PET in Squamous Cell Carcinoma of the Head and Neck

Rui-Yun Chen, Ying-Chun Lin, Wei-Chih Shen, Te-Chun Hsieh, Kuo-Yang Yen, Shang-Wen Chen, Chia-Hung Kao

Appendix 1. Representative images of IHC staining for PD-L1. A) negative PD-L1 staining, B) weak membranous staining, C) moderate membranous staining, D) strong membranous staining.

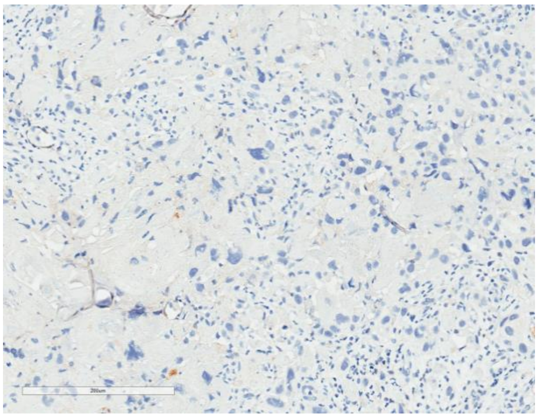	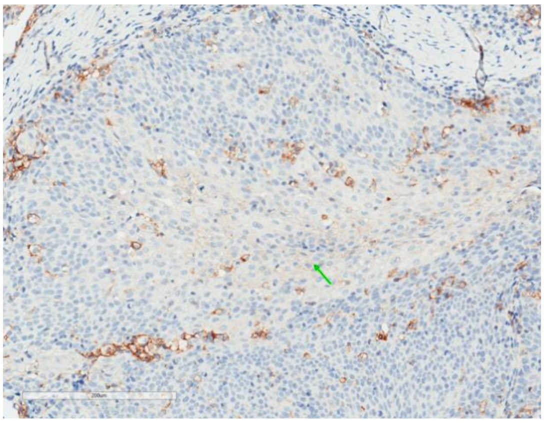
A: Negative PD-L1 staining. (original magnification: 200×)	B: Weak membranous staining of neoplastic cells (original magnification: 200×)
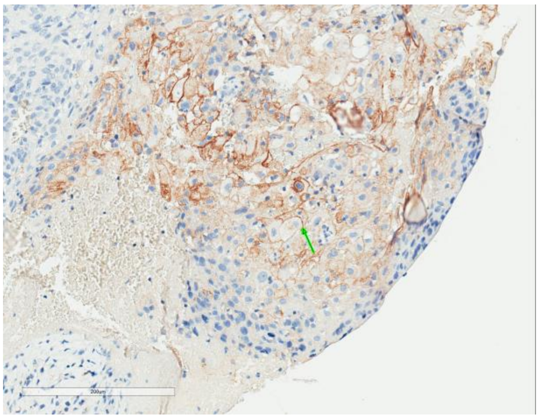	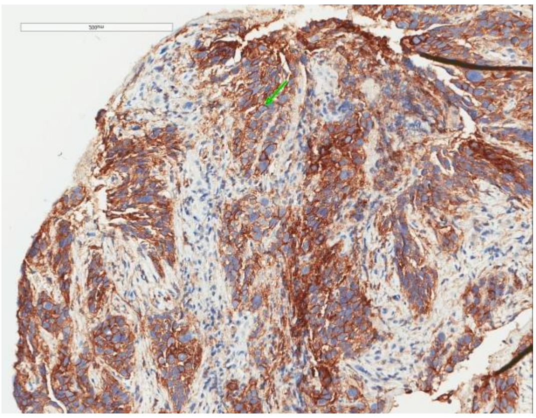
C: Moderate membranous staining of neoplastic cells (original magnification: 200×)	D: Strong membranous staining of neoplastic cells (original magnification: 200×)

Appendix 2. Representative images of *p16* positive cells. A) negative PD-L1 staining, B) only faintly cytoplasmic staining, < 70%, C) weakly cytoplasmic staining, D) moderate cytoplasmic staining, E) diffusely cytoplasmic and focal nuclear, F) Diffusely cytoplasmic and nuclear staining.

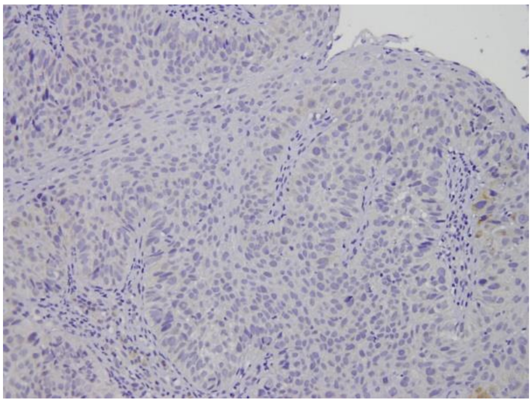	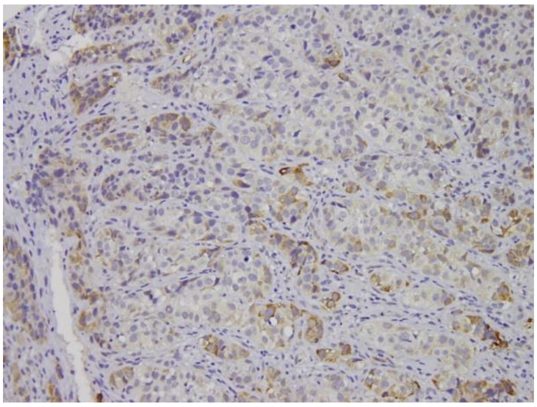
A. Negative staining (original magnification 200x)	B. Only faintly cytoplasmic staining, < 70% (original magnification 200x)
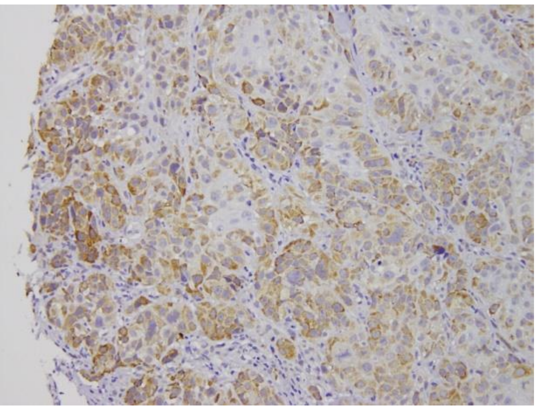	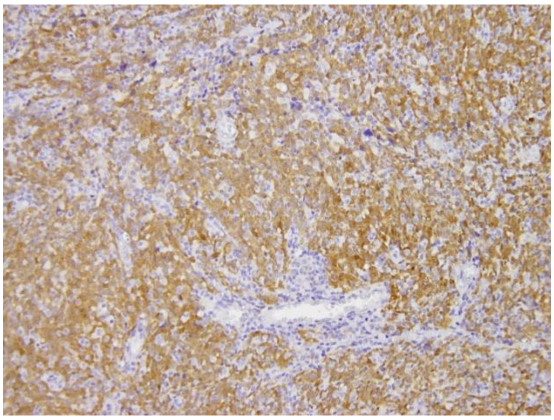
C. Weakly cytoplasmic staining, > 70% (original magnification 200x)	D. Moderately cytoplasmic staining, > 70% (original magnification 200x)
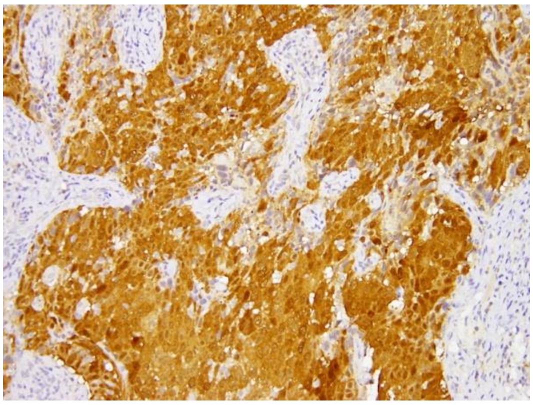	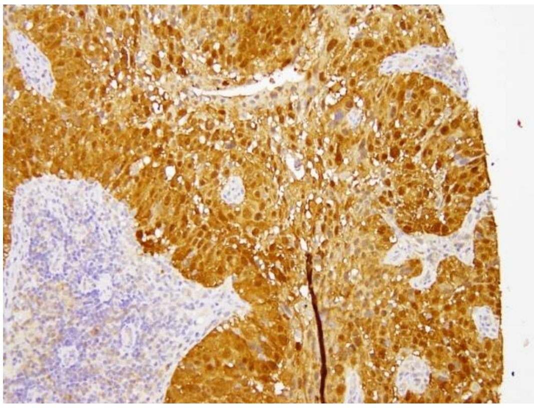
E. Diffusely cytoplasmic and focal nuclear staining (original magnification 200x)	F. Diffusely cytoplasmic and nuclear staining (original magnification 200x)

Appendix 3. Indices calculated from textural analysis and the area under the ROC curve for predicting PD-L1 expression and immunohistochemical biomarkers.

Classification of matrix	Index
Conventional PET-related parameter	SUVmax
	MTV
	TLGmean
Histogram	Variance
	Skewness
	Kurtosis
	Peak
Gray Level Cooccurrence Matrix (GLCM)	Autocorrelation
	Homogeneity
	Energy
	Correlation
	Contrast
	Entropy
	Dissimilarity
Gray-Level Run Length Matrix (GLRLM)	SRE
	LRE
	LGRE
	HGRE
	SRLGE
	SRHGE
	LRLGE
	LRHGE
	GLNUr
	RLNU
	RP
Neighborhood Gray-Level Different Matrix (NGLDM)	Coarseness
	Contrast
	Busyness
	Complexity
	Strength
Gray-Level Zone Length Matrix (GLSZM)	SZE
	LZE
	LGZE
	HGZE
	SZLGE

SZHGE
LZLGE
LZHGE
GLNUz
ZLNU
ZP

Abbreviations: SRE = short-run emphasis; LRE = long-run emphasis; LGRE = low gray-level run emphasis; HGRE = high gray-level run emphasis; SRLGE = short-run low gray-level emphasis; SRHGE = short-run high gray-level emphasis; LRLGE = long-run low gray-level emphasis; LRHGE = long-run high gray-level emphasis; GLNUr = gray-level nonuniformity for run; RLNU = run length nonuniformity; RP = run percentage; SZE = short-zone emphasis; LZE = long-zone emphasis; LGZE = low gray-level zone emphasis; HGZE = high gray-level zone emphasis; SZLGE= short-zone low gray-level emphasis; SZHGE = short-zone high gray-level emphasis; LZLGE = long-zone low gray-level emphasis; LZHGE = long-zone high gray-level emphasis; GLNUz = gray-level nonuniformity for zone; ZLNU = zone length nonuniformity; and ZP = zone percentage.

Appendix 4. Correlation between IHC percentage and textural features using Spearman's correlation (*p* value/correlation coefficiency)

Classification	Index	*HIF-1α*	*VEGF*	*CAIX*	*Glut1*	*Ki-67*	*c-Met*	*Claudin-4*	*Bcl-2*	*p16*
CT-based volume	GTV		0.028/0.29							
Classical PET	SUVmax					0.026/0.30	0.011/-0.34	0.006/-0.36		
	MTV									
	TLGmean								0.046/-0.28	
Histogram	variance						0.008/-0.36	<0.001/-0.53		
	skewness									
	kurtosis									
	peak		0.031/0.03			0.04/0.28		0.015/-0.33		0.049/0.27
GLCM	homogeneity						0.006/0.37	<0.001/0.65		
	energy						0.008/0.35	<0.001/0.53	0.034/0.29	
	correlation									
	contrast			0.04/0.28			0.005/-0.38	<0.001/-0.57		
	entropy	0.04/0.28					0.008/-0.35	<0.001/-0.52		
	dissimilarity						0.005/-0.38	<0.001/-0.59		
	autocorrelation									
GLRLM	SRE									
	LRE									
	LGRE		0.018/-0.32			0.012/-0.34				0.037/-0.29
	HGRE		0.02/0.31			0.012/0.33		0.023/-0.30		
	SRLGE		0.021/-0.31			0.013/-0.33				0.034/-0.29

	SRHGE		0.02/0.31			0.013/0.33		0.024/-0.30		
	LRLGE		0.03/-0.29			0.006/-0.36				
	LRHGE		0.015/0.32			0.014/0.33		0.023/-0.30		0.04/0.28
	GLNUr	0.001/0.23								
	RLNU	0.02/0.31								
	RP									
NGLDM	coarseness	0.026/-0.30						0.04/0.27		
	contrast								0.04/0.27	
	busyness						0.002/0.41	0.001/0.43		
	complexity			0.029/0.29			0.001/-0.45	<0.001/-0.52		
	strength						<0.001/-0.49	<0.001/-0.51		
GLSZM	SZE							0.008/-0.35		
	LZE							0.006/0.36		
	LGZE		0.023/-0.30			0.014/-0.33				
	HGZE		0.032/0.29			0.02/0.31		0.02/-0.31		
	SZLGE					0.012/-0.33				0.022/-0.31
	SZHGE			0.018/0.31		0.013/0.29		0.007/-0.36		
	LZLGE					0.028/-0.29		0.012/0.33		
	LZHGE									
	GLNUz	0.03/0.29	0.02/0.31							
	ZLNU				0.047/0.28			0.001/-0.42		
	ZP							<0.001/-0.53		

Note: 1. Abbreviations: as Appendix 3.

2. No correlation was observed between textural features and *YAP1* or *EGFR*.

Appendix 5. Quantitative textural indices statistically associated with the intensity of biomarkers according to the optimal cutoffs or 50% percentile of immunohistochemical score

Variables	Volumetric factors or textural indices
HIF-1α stain percentage	
<80% vs. ≥80%	GTV
<50% vs. ≥50% percentile	Entropy(GLCM), Coarseness, GLNUz, RLNU
*VEG*F	
IRS score 0-2 vs. 3-12	GTV, MTV, TLGmean, RLNU, GLNUz, Energy*
<50% vs. ≥50% percentile	GTV, SUVmax, TLGmean, Entropy, Energy*, Contrast, Dissimilarity, Homogeneity*, Complexity, Strength, LGRE*, HGRE,
	SRLGE*, SRHGE, LRLGE*, LRHGE, LZE*, ZP, ZLNU, LGZE*, HGZE, SZHGE, SZLGE*, LZLGE*
GLUT-1 stain percentage	
<90% vs. ≥90%	MTV, TLGmean, ZLNU*
<50% vs. ≥50% percentile	SUVmax, TLGmean, Contrast, Dissimilarity, Homogeneity*, LGRE*, HGRE, SRLGE*, SRHGE, LRLGE*, LRHGE, SZE,
	ZLNU, LGZE, HGZE, SZHGE, SZLGE*, LZLGE*
Claudin-4 ,	
IRS score 0-4 vs. 5-12	Variance*, Homogeneity, Energy, Entropy*, Busyness, SZE*, LZE, HGZE*, SZHGE, LZLGE, ZP*, ZLNU*
<50% vs. ≥50% percentile	SUVmax*, Variance*, Contrast*, Dissimilarity*, Energy, Entropy*, Homogeneity, Coarseness, Busyness, HGRE*, SRHGE*,
LRHGE*,	
	SZE*, LZE, GLNUz*, RLNU*, RLNU*ZP*, ZLNU*, HGZE*, SZHGE*, LZLGE
CAIX stain percentage	
<10% vs. ≥10%	none
<15% vs. ≥15%	none
<30% vs. ≥30%	Variance*, Homogeneity

<35% vs. ≥35%	none
<50% ≥50% percentile	none
c-Met stain intensity	,
negative + weak vs. strong	SUVmax*, Entropy*, Energy, SRE, LRE*, LRHGE*, LZHGE*
<50% vs. ≥50% percentile	SUVmax*, Variance*,Contrast*, Dissimilarity*, Homogeneity, Busyness, HGRE*, SZHGE*, HGZE*,SRHGE*, LRHGE*, ,
Bcl-2 stain	
<50% vs. ≥50% percentile	none
YAP-1 positive stain percentage	
<50% vs. ≥50% percentile	none
Ki-67 stain percentage	
< 15% vs. ≥15%	SUVmax, SRE, LRE*, HGRE, SRLGE*, SRHGE, LRLGE*, LRHGE, LGZE*, HGZE, SZLGE*
<50% vs. ≥50% percentile	SUVmax, SRE, LRE*, LGRE*, HGRE, SZHGE, SRLGE*, SRHGE, LRHGE, LGZE*, HGZE, SZLGE*, LRLGE*,
EGFR	
< 65% vs. ≥65%	
<50% vs. ≥50% percentile	none
p16 stain percentage	
< 70% vs. ≥70%	none

Abbreviation: IRS = immunoreactive score

Note: 1. Asterisk represented an inverse correlation.

2. The optimal cutoffs were reported in previous study [7].
